# Clinical and laboratory profiles of acute central nervous system infections in a Chinese pediatric cohort: a five-year retrospective analysis

**DOI:** 10.1186/s12887-026-06932-1

**Published:** 2026-04-25

**Authors:** Shuyun Wang, Peibin Hou, Yu Ma, Xin Lv, Wandong Hu, Qian Zeng, Lingdong Zhu

**Affiliations:** 1 Clinical Laboratory, Children’s Hospital Affiliated to Shandong University, Jinan, China; 2 Clinical Laboratory, Jinan Children’s Hospital, Jinan, China; 3 Shandong Center for Disease Control and Prevention, Jinan, China; 4 Disease Prevention and Control Center of China Railway Jinan Group Co Ltd, Jinan, China; 5 Department of Pediatric Neurology, Children’s Hospital Affiliated to Shandong University, Jinan, China

**Keywords:** Central nervous system infections, Children, Bacterial CNS infections, Nonbacterial CNS infections, Pathogen, Pediatric intensive care unit, Retrospective analysis

## Abstract

**Background:**

Acute central nervous system (CNS) infections in children cause severe neurological morbidity, but early diagnosis and risk assessment remain challenging. This study aimed to characterize the clinical and laboratory features, pathogen spectrum, and risk factors for severe disease in a five-year pediatric cohort.

**Methods:**

We retrospectively analyzed children diagnosed with acute CNS infections at the Shandong Regional Children’s Medical Center from January 2020 to December 2024. Clinical, laboratory, and outcome data were extracted from electronic medical records.

**Results:**

Among 422 enrolled children, 132 (31.3%) had clinically diagnosed bacterial and 290 (68.7%) nonbacterial CNS infections. Bacterial infections were associated with significantly younger age (median 0.22 vs. 4.02 years), longer hospitalization (23.5 vs. 13 days), and higher rates of systemic complications (all *P* < 0.001). To distinguish bacterial from nonbacterial CNS infections, ROC analysis demonstrated that CSF protein had excellent discriminative ability (AUC = 0.917), followed by serum albumin (AUC = 0.869) and CSF white blood cells (AUC = 0.837). CRP (AUC = 0.801) and PCT (AUC = 0.682) were also evaluated. A total of 175 children (41.5%) required pediatric intensive care unit (PICU) admission. In addition to impaired consciousness, multivariate analysis identified respiratory failure (aOR = 39.76), somnolence (aOR = 15.03), vomiting (aOR = 3.93), seizure (aOR = 2.53), pneumonia (aOR = 2.51), age (aOR = 1.09), and length of hospital stay (aOR = 1.02) as independent predictors of PICU admission (all *P* < 0.05).

Pathogens were identified in 94 cases (54 bacterial and 40 viral). Among bacterial pathogens, Streptococcus pneumoniae (*n*=13) and Escherichia coli (*n*=10) predominated, with 68.5% (37/54) occurring in infants ≤1 year. mNGS detected pathogens missed by conventional culture and identified co-infections.

**Conclusions:**

In addition to CSF analysis, CRP can help distinguish bacterial from nonbacterial CNS infections; serum albumin and total protein (~50% sensitivity) can also help but should not be used alone. Impaired consciousness, respiratory failure, somnolence, vomiting, seizure, and pneumonia were key risk factors for PICU admission.The relatively high rate of bacterial infections in infants warrants particular clinical attention. These findings can guide clinical practice and improve outcomes in this vulnerable population.

**Supplementary Information:**

The online version contains supplementary material available at 10.1186/s12887-026-06932-1.

## Introduction

Acute central nervous system (CNS) infections are a major global threat to children’s health, encompassing life-threatening conditions such as meningitis, encephalitis, and meningoencephalitis that require urgent care. Without timely and appropriate treatment, these infections can lead to severe outcomes including high morbidity, fatal complications, and long-term neurological sequelae, especially in low- and middle-income countries [1, 2].

Clinically, acute CNS infections in children are caused by a wide range of microorganisms, predominantly viruses and bacteria, as well as fungi and parasites [3]. Over the past few decades, vaccines against *Haemophilus influenzae* (*H. influenzae*) type b, *Streptococcus pneumoniae* (*S. pneumoniae*) and *Neisseria meningitidis* (*N. meningitidis*) have effectively reduced morbidity and mortality from childhood bacterial meningitis in many regions. Nevertheless, CNS infections continue to pose substantial public health challenges [4]. Bacterial CNS infections are particularly aggressive and potentially fatal without prompt antimicrobial therapy. Early differentiation between clinical bacterial and nonbacterial CNS infections is therefore critically important to guide timely treatment and improve outcomes.

However, accurate early distinction remains clinically challenging. Routine diagnosis of bacterial CNS infection mainly depends on cerebrospinal fluid (CSF) examinations, including positive CSF culture, the traditional “gold standard,” or CSF analysis (elevated white blood cell count, protein level, and decreased glucose concentration). Nonetheless, prior antibiotic use and early disease stage often lead to negative cultures or even normal CSF profiles, limiting its diagnostic value [5]. Although conventional molecular methods such as polymerase chain reaction (PCR) and multiplex PCR panels are now widely used in clinical diagnostics [6], metagenomic next-generation sequencing (mNGS) has further improved pathogen detection in complex CNS infections [7]. However, its widespread application is currently restricted by high costs and specialized requirements, especially in developing regions.

In cases where lumbar puncture is medically contraindicated, blood-derived nonspecific inflammatory biomarkers such as procalcitonin (PCT) and C-reactive protein (CRP) provide accessible and rapid supportive evidence for bacterial infection [8]. Consequently, comprehensive evaluation of clinical manifestations, laboratory test results, and microbiological examination findings is critically important, as it can provide clinical clues and facilitate rapid differentiation of pathogens. Furthermore, early identification of children at risk of clinical deterioration is crucial for timely intensive care and improved prognosis.

Few studies have focused on pediatric CNS infections in North China, and large multi-year cohort studies are essential to guide clinical practice. To address these gaps, this study analyzed a five-year cohort of pediatric CNS infection patients in North China with three objectives: (1) to delineate the pathogenic spectrum of pediatric CNS infections; (2) to characterize clinical and laboratory differences between clinical bacterial and nonbacterial infections; and (3) to identify risk factors predictive of pediatric intensive care unit (PICU) admission. This comprehensive evaluation aims to optimize diagnostic precision, guide therapeutic decisions, and improve clinical outcomes for the pediatric population.

## Methods

### Study design and population

This retrospective study was conducted at the Children’s Hospital Affiliated to Shandong University (CHASU) /Shandong Regional Children’s Medical Center between January 1, 2020, and December 31, 2024. As the largest tertiary pediatric hospital in Shandong Province (1,400 beds) and the designated regional children’s medical center, the institution serves more than 56,000 inpatients and 1.2 million outpatients annually. Shandong covers an area of 157,780 km² with a population of 101.23 million, ranking as the second most populous province in China.

Patients were included according to the following criteria: (i) Age from 1 month to < 18 years; (ii) Clinical diagnosis of encephalitis, meningoencephalitis, or meningitis based on established criteria [9, 10]; (iii) Availability of complete clinical and laboratory data.

Exclusion criteria were as follows: (i) Presence of underlying medical conditions, including but not limited to leukemia, congenital heart disease, immunodeficiency disorders or autoimmune diseases (e.g., autoimmune encephalitis); (ii) Status following traumatic cranial surgery; (iii) Identification as a secondary referral case during the same illness episode; (iv) Clinically diagnosed fungal or parasitic CNS infections.

In this study, patients were categorized into two etiological groups: bacterial CNS infections, based on specific criteria, and nonbacterial CNS infections, which included clinically diagnosed viral infections and cases of unknown etiology. Clinical bacterial CNS infection was diagnosed based on a combination of clinical features (such as acute fever, irritability, altered mental status, headache, vomiting, seizures, and meningeal signs) and CSF findings (pleocytosis > 100 × 10⁶/L with neutrophil predominance, elevated protein > 1,000 mg/L, decreased glucose < 2.2 mmol/L) [10, 11]. Confirmed bacterial CNS infection was defined by positive CSF or blood culture, or positive molecular detection (e.g., PCR, mNGS) from CSF. Nonbacterial CNS infections included clinical viral CNS infections and cases with unknown etiology, and did not include clinically diagnosed fungal or parasitic CNS infections.

### Data collection

In this study, demographic and clinical data were systematically collected from the hospital information system (HIS). Extracted information included age, sex, duration of hospitalization, admission to the PICU, and presenting symptoms such as fever, headache, vomiting, seizure, rash, cough, somnolence, impaired consciousness, neck stiffness, jaundice, and gait disturbance. Laboratory parameters for blood and CSF analyses were obtained from the Hospital Health Medical Big Data Research and Innovation Platform. Blood tests included WBC count, neutrophil count, lymphocyte count, platelet count, CRP, PCT, total protein, albumin, glucose, and lactate dehydrogenase. CSF analysis encompassed measurements of protein, glucose, chloride, WBC count, segmented neutrophil count, and monocyte count.

Neuroimaging findings, including computed tomography, magnetic resonance imaging, and transfontanelle ultrasonography, were documented to assess for hydrocephalus, encephalomalacia, subdural effusion, and non-traumatic intracranial hemorrhage. Other complications recorded during hospitalization included sepsis, pneumonia, anemia, hand, foot and mouth disease, herpangina, and respiratory failure. Patients with missing essential data were excluded from the analysis.

### Microbiological methods

For bacterial detection, CSF culture was performed in 419 of 422 patients (99.3%). The three patients who did not undergo CSF culture were clinically diagnosed with viral CNS infections and all had negative blood cultures. CSF specimens were cultured on Columbia blood agar and chocolate agar. Blood culture was performed in 393 of 422 patients (93.1%) using aerobic and anaerobic blood culture bottles.

For viral detection, serological testing for Japanese encephalitis virus (JEV) and herpes simplex virus (HSV) was performed as clinically indicated.

In addition, mNGS was performed for pathogen detection in clinically challenging cases, such as negative cultures despite high suspicion, severe or atypical presentations, or suspected rare pathogens. In our cohort, 59 of 422 patients (14.0%) underwent mNGS, including 38 patients with bacterial CNS infections (31 with DNA sequencing alone, 7 with both DNA and RNA sequencing) and 21 patients with viral CNS infections (7 with DNA sequencing alone, 9 with RNA sequencing alone, 5 with both).

For mNGS, CSF samples were used for nucleic acid extraction using the QIAamp UCP Pathogen Mini Kit (Qiagen, Germany) for DNA and the QIAamp Viral RNA Mini Kit (Qiagen) for RNA, following the manufacturers’ instructions. cDNA was generated using reverse transcriptase and dNTPs (Thermo Fisher). Libraries were constructed using the KAPA Low Throughput Library Construction Kit (KAPA Biosystems, U.S.A.) following the manufacturer’s protocol. Library quality was assessed using the Qubit dsDNA HS Assay Kit followed by the High Sensitivity DNA Kit (Agilent) on an Agilent 2100 Bioanalyzer. Library pools were then loaded onto an Illumina Nextseq CN500 sequencer for 75 cycles of single-end sequencing to generate approximately 20 million reads for each library.

Bioinformatic analysis of mNGS was performed as follows. Trimmomatic was used to remove low-quality reads, duplicates, adapters, and reads shorter than 70 bp. Low-complexity reads were removed using Kcomplexity. Human sequence data were removed by alignment to the hg38 reference genome using SNAP v1.0beta.18. The microbial genome database was constructed using representative assemblies from NCBI based on Kraken 2 criteria (https://benlangmead.github.io/aws-indexes/k2). Microbial reads were aligned to the database using Burrows-Wheeler Aligner, and reads with 90% identity of reference were defined as mapped reads. Reads with multiple alignments within the same genus were excluded, and only reads mapped to the same species were considered. The clinical reportable range was established according to three sources referenced in a previous study [12] : the Johns Hopkins ABX Guide (https://www.hopkinsguides.com/hopkins/index/Johns_Hopkins_ABX_Guide/Pathogens), the Manual of Clinical Microbiology, and peer-reviewed case reports. For viruses, bacteria, and parasites, a species-level microbe was confirmed if its coverage or pathogenicity was at least 10-fold higher than any other microbe in the sample. For quality control, a negative control and sterile deionized water were processed with clinical samples in each batch.

### Statistical analysis

Data analysis was performed using SPSS (Version 26.0). Categorical variables are presented as numbers (percentages) and compared using the Chi-square test or Fisher’s exact test, as appropriate. The distribution of continuous variables was assessed for normality. Normally distributed variables are expressed as mean ± standard deviation and compared using the Student’s t-test. Non-normally distributed variables are reported as median (interquartile range, IQR) and compared using the Mann-Whitney U test for comparisons between two groups, or the Kruskal-Wallis H test for multiple-group comparisons. Statistical significance was determined using a threshold of *P* < 0.05.

To adjust for multiple comparisons, Benjamini-Hochberg false discovery rate (FDR) correction was applied to the comparative tables. Adjusted *P* values (*q* values) are reported in the tables, and a threshold of q < 0.05 was considered statistically significant after correction.

To evaluate the diagnostic performance of biomarkers for distinguishing bacterial from nonbacterial CNS infections, receiver operating characteristic (ROC) analysis was performed. The area under the curve (AUC), 95% confidence intervals (CI), and *P* values were calculated for each biomarker. Optimal cut-off values were determined using the Youden index.

To identify independent predictors of PICU admission, binary logistic regression analysis was performed with PICU admission as the dependent variable. Candidate variables with *P* < 0.05 in univariate analysis and age were entered as covariates. Adjusted odds ratios (aOR) with 95% confidence intervals (CI) were calculated. Variables with complete separation were excluded from the model. Model fit was assessed using the Hosmer-Lemeshow test. Multicollinearity was assessed using the variance inflation factor (VIF), with a threshold of 5 indicating significant multicollinearity.

To assess the robustness of our findings, sensitivity analyses were performed: (1) excluding preterm neonates (*n* = 13), with comparisons between bacterial and nonbacterial groups re-analyzed; and (2) analyzing annual case distribution to assess potential confounding by the COVID-19 pandemic. All subgroup analyses were conducted as post hoc and exploratory and were not pre-specified.

## Results

### Demographic and clinical characteristics

**Fig. 1:**
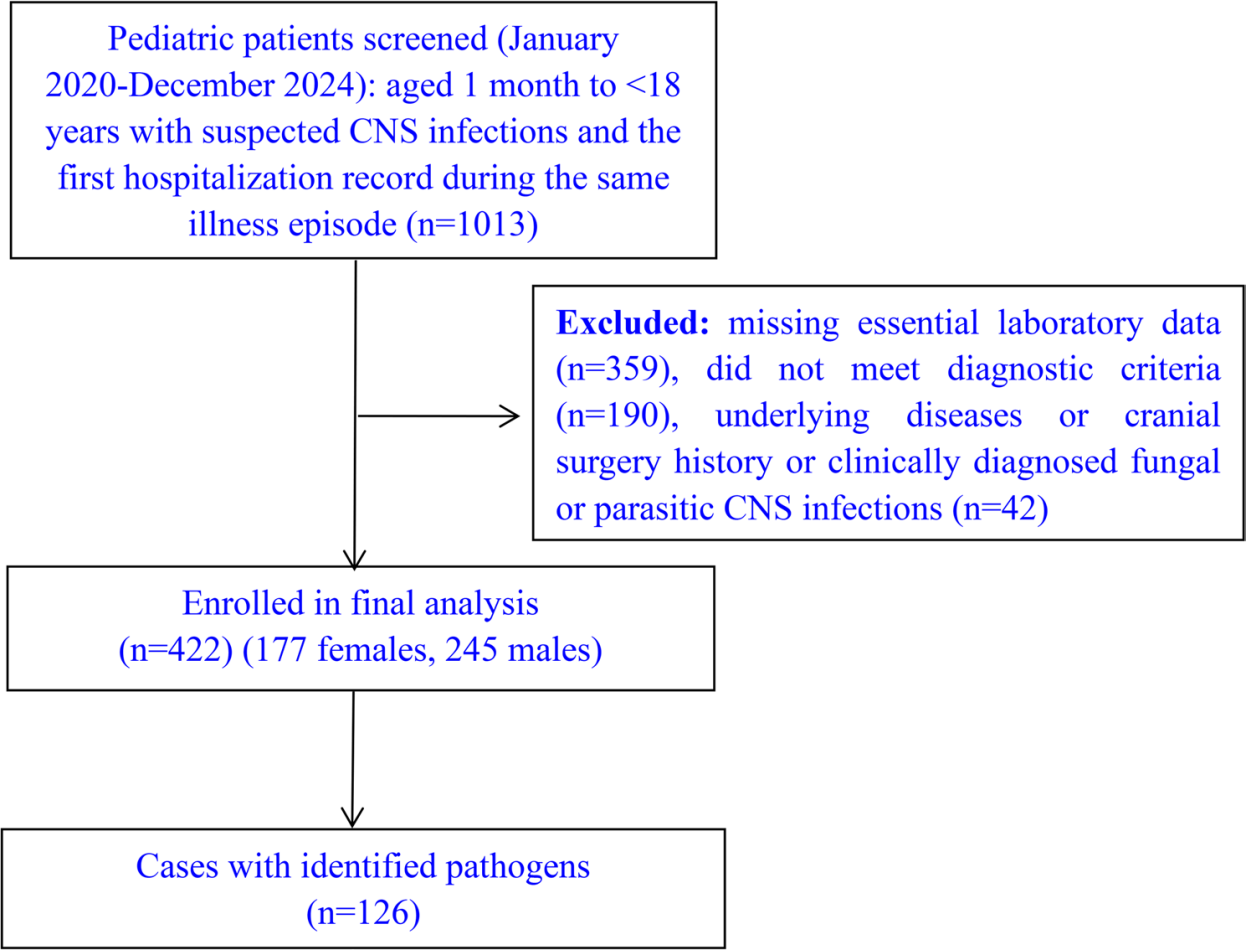
Flow diagram for subject selection in a retrospective cohort study of central nervous system infections in children in Shandong Province, 2020–2024

Between January 2020 and December 2024, a total of 1013 pediatric patients (age 1 month to < 18 years) with suspected CNS infections and the first hospitalization record during the same illness episode were screened (Fig. 1). Based on the inclusion and exclusion criteria, 591 patients were excluded: 190 who did not meet the diagnostic criteria for CNS infection (suspected but not confirmed cases under observation), 359 with missing essential laboratory data, and 42 with underlying diseases or cranial surgery history. Among the 359 patients excluded due to missing data, the most frequently missing variable was procalcitonin (PCT) (85 cases, 23.7%). The excluded patients were predominantly nonbacterial cases (325/359, 90.5%) and had a low proportion of PICU admission rate (24/359, 6.7%), suggesting that patients with milder symptoms were more likely to have incomplete data. This pattern reflects clinical practice, where PCT testing is not routinely performed in all patients.

Finally, 422 patients (177 females, 245 males) were enrolled and classified into three diagnostic groups: encephalitis (*n* = 274, 64.9%), meningoencephalitis (*n* = 17, 4.0%), and meningitis (*n* = 131, 31.0%). The annual distribution of admissions did not differ significantly among groups (*P* = 0.411). The median length of hospital stay for the entire cohort was 15 days and varied significantly by diagnosis (*P* < 0.001). It was shortest in patients with encephalitis (13 days), compared with those with meningoencephalitis or meningitis (23 days each). Similarly, the overall median age was 2.38 years and differed significantly across groups (*P* < 0.001). Patients with encephalitis were the oldest (median 3.89 years), whereas those with meningitis and meningoencephalitis were markedly younger (median 0.23 and 0.18 years, respectively). Consistent with this age distribution, meningoencephalitis and meningitis occurred predominantly in infants (≤ 1 year: 94.1% and 70.2%, respectively), while encephalitis was observed primarily in children older than 1 year (94.2%) (Table 1).

**Table 1:** Demographic and clinical characteristics of patients in this study with encephalitis, meningoencephalitis and meningitis

Characteristics	All (*n* = 422)	Encephalitis (*n* = 274)	Meningoencephalitis (*n* = 17)	Meningitis (*n* = 131)	*P* Value	Adjusted *P* Value*
Male Sex, *n* (%)	245 (58.1)	162 (59.1)	7 (41.2)	76 (58.0)	0.347ᵃ	0.496
Length of Hospital Stay, days, Median (IQR)	15.00 (10.00, 22.00)	13.00 (9.00, 16.00)	23.00 (13.50, 41.00)	23.00 (16.00, 31.00)	< 0.001ᶜ	< 0.05
Age in years, Median (IQR)	2.38 (0.63, 5.52)	3.89 (1.92, 6.58)	0.18 (0.11, 0.50)	0.23 (0.12, 1.45)	< 0.001ᶜ	< 0.05
Age Group, *n* (%)					< 0.001ᶜ	< 0.05
≤ 1 year	124 (29.4)	16 (5.8)	16 (94.1)	92 (70.2)		
> 1–3 years	112 (26.5)	99 (36.1)	0 (0.0)	13 (9.9)		
> 3–5 years	66 (15.6)	53 (19.3)	0 (0.0)	13 (9.9)		
> 5–7 years	54 (12.8)	50 (18.2)	0 (0.0)	4 (3.1)		
> 7–10 years	35 (8.3)	32 (11.7)	0 (0.0)	3 (2.3)		
> 10 years	31 (7.3)	24 (8.8)	1 (5.9)	6 (4.6)		
Admission Year, *n* (%)					0.411ᵇ	0.514
2020	92 (21.8)	59 (21.5)	3 (17.6)	30 (22.9)		
2021	131 (31.0)	90 (32.8)	2 (11.8)	39 (29.8)		
2022	69 (16.4)	43 (15.7)	5 (29.4)	21 (16.0)		
2023	89 (21.1)	60 (21.9)	4 (23.5)	25 (19.1)		
2024	41 (9.7)	22 (8.0)	3 (17.6)	16 (12.2)		
Clinical Bacterial CNS Infection, *n* (%)	132 (31.3)	0 (0.0)	11 (64.7)	121 (92.4)	< 0.001ᵇ	< 0.05
PICU Admission, *n* (%)	175 (41.5)	122 (44.5)	7 (41.2)	46 (35.1)	0.198ᵃ	0.330
Prognosis, *n* (%)						
Cured	301 (71.3)	197 (71.9)	11 (64.7)	93 (71.0)	0.785ᵇ	0.785
Improved	95 (22.5)	57 (20.8)	4 (23.5)	34 (26.0)	0.489ᵇ	0.543
Not healed	26 (6.2)	20 (7.3)	2 (11.8)	4 (3.1)	0.105ᵇ	0.210

*IQR* Interquartile range, *CNS* Central nervous system, *PICU* Pediatric intensive care unit

Categorical variables are presented as *n* (%). Continuous variables not following a normal distribution are presented as median (interquartile range)

*P* values are for comparisons across the encephalitis, meningoencephalitis, and meningitis groups

Values < 0.05 were considered statistically significant after correction

Statistical Methods: ᵃChi-square test; ᵇFisher’s exact test; ᶜKruskal-Wallis H test

*Adjusted *P* values were calculated using the Benjamini-Hochberg FDR method

The prevalence of clinical bacterial infections differed significantly across diagnostic groups (*P* < 0.001). Meningitis was predominantly bacterial (92.4%), whereas all encephalitis cases were nonbacterial. Meningoencephalitis had a mixed etiology, predominantly bacterial (64.7%). The overall PICU admission rate was 41.5%, with no significant intergroup difference (*P* = 0.198). The overall clinical outcomes were favorable, with a combined cure and improvement rate of 93.9%. The distribution of specific outcomes (cured, improved, not healed) showed no significant differences across diagnostic groups (all *P* > 0.05), and no fatalities were documented in any group (Table 1).

### Clinical bacterial and nonbacterial CNS infections

Of the 422 patients with CNS infections, 132 (31.3%) were clinically diagnosed as bacterial and 290 (68.7%) as nonbacterial (Table 2). Patients with clinical bacterial infections were significantly younger (median 0.22 years) than those with clinical nonbacterial infections (median 4.02 years; *P* < 0.001). In total, 78.0% of clinical bacterial cases occurred in infants aged ≤ 1 year, whereas 92.8% of clinical nonbacterial cases were in children older than 1 year (*P* < 0.001). The median hospitalization was also significantly longer for clinical bacterial cases (23.5 days) than for nonbacterial cases (13 days; *P* < 0.001). No significant difference was observed in sex distribution or PICU admission rates between the two groups (*P* = 0.231 and *P* = 0.711, respectively).

**Table 2:** Demographic, clinical and laboratory findings in children clinically diagnosed with bacterial and nonbacterial central nervous system infections

Characteristics	Clinical Bacterial CNS Infection (*n* = 132)	Clinical Nonbacterial CNS Infection (*n* = 290)	*P* Value	Adjusted *P* Value*
Male Sex, *n* (%)	71 (53.8)	174 (60.0)	0.231ᵃ	0.304
Length of Hospital Stay, days, Median (IQR)	23.50 (18.25, 33.75)	13.00 (9.75, 16.00)	< 0.001^d^; Z=-11.326	< 0.05
Age in years, Median (IQR)	0.22 (0.12, 0.79)	4.02 (1.96, 6.99)	< 0.001^d^; Z=-12.882	< 0.05
Age Group, *n* (%)			< 0.001ᵃ	< 0.05
≤ 1 year	103 (78.0)	21 (7.2)		
> 1–3 years	12 (9.1)	100 (34.5)		
> 3–5 years	11 (8.3)	55 (19.0)		
> 5–7 years	2 (1.5)	52 (17.9)		
> 7–10 years	1 (0.8)	34 (11.7)		
> 10 years	3 (2.3)	28 (9.7)		
PICU Admission, *n* (%)	53 (40.2)	122 (42.1)	0.711ᵃ	0.779
Clinical characteristics, *n* (%)				
Fever	122 (92.4)	261 (90.0)	0.425ᵃ	0.514
Headache	5 (3.8)	47 (16.2)	< 0.001ᵃ	< 0.05
Seizure	25 (18.9)	142 (49.0)	< 0.001ᵃ	< 0.05
Vomiting	11 (8.3)	32 (11.0)	0.395ᵃ	0.491
Rash	13 (9.8)	45 (15.5)	0.117ᵃ	0.163
Cough	15 (11.4)	30 (10.3)	0.753ᵃ	0.770
Somnolence	5 (3.8)	13 (4.5)	0.743ᵃ	0.777
Impaired Consciousness	2 (1.5)	37 (12.8)	< 0.001ᵇ	< 0.05
Neck Stiffness	4 (3.0)	2 (0.7)	0.080ᵇ	0.127
Jaundice	5 (3.8)	7 (2.4)	0.528ᵇ	0.592
Gait Disturbance	0 (0.0)	7 (2.4)	0.104ᵇ	0.150
Sepsis	92 (69.7)	16 (5.5)	< 0.001ᵃ	< 0.05
Pneumonia	69 (52.3)	92 (31.7)	< 0.001ᵃ	< 0.05
Anemia	35 (26.5)	12 (4.1)	< 0.001ᵃ	< 0.05
Hand, Foot and Mouth Disease	0 (0.0)	30 (10.3)	< 0.001ᵇ	< 0.05
Herpangina	0 (0.0)	42 (14.5)	< 0.001ᵇ	< 0.05
Prematurity	12 (9.1)	1 (0.3)	< 0.001ᵇ	< 0.05
Respiratory Failure	9 (6.8)	26 (9.0)	0.458ᵃ	0.540
Hydrocephalus	16 (12.1)	0 (0.0)	< 0.001ᵇ	< 0.05
Encephalomalacia	7 (5.3)	0 (0.0)	< 0.001ᵇ	< 0.05
Subdural Effusion	2 (1.5)	0 (0.0)	0.097ᵇ	0.149
Non-traumatic Intracranial Hemorrhage	2 (1.5)	0 (0.0)	0.097ᵇ	0.144
Prognosis, *n* (%)				
Cured	91 (68.9)	210 (72.4)	0.464^a^	0.534
Improved	35 (26.5)	60 (20.7)	0.184^a^	0.249
Not healed	6 (4.5)	20 (6.9)	0.352^a^	0.450
Blood Parameters, Median (IQR)				
White Blood Cell (×10⁹/L)	11.80 (9.00, 16.40)	8.62 (6.13, 11.59)	< 0.001^d^; Z=-6.197	< 0.05
Neutrophil (×10⁹/L)	6.01 (3.24, 10.41)	4.70 (2.71, 7.93)	0.019^d^; Z=-2.341	0.031
Lymphocyte (×10⁹/L)	3.80 (2.21, 5.75)	2.21 (1.34, 3.67)	< 0.001^d^; Z=-5.957	< 0.05
Platelet (×10⁹/L)	373.00 (233.50, 514.00)	274.50 (218.75, 357.25)	< 0.001^d^; Z=-5.040	< 0.05
C-reactive protein (mg/L)	36.70 (3.36, 97.27)	3.20 (1.40, 8.33)	< 0.001^d^; Z=-9.909	< 0.05
Procalcitonin (ng/mL)	0.34 (0.10, 3.93)	0.13 (0.06, 0.33)	< 0.001^d^; Z=-5.985	< 0.05
Total Protein (g/L)	54.90 (48.65, 59.30)	64.00 (59.80, 68.60)	< 0.001^d^; Z=-10.457	< 0.05
Albumin (g/L)	34.60 (31.90, 37.40)	41.05 (38.28, 43.10)	< 0.001^d^; Z=-12.169	< 0.05
Glucose (mmol/L)	5.33 (4.70, 6.40)	5.39 (4.76, 6.32)	0.756^d^; Z=-0.311	0.756
Lactate dehydrogenase (U/L)	251.00 (202.50, 294.75)	249.00 (210.00, 307.00)	0.730^d^; Z=-0.346	0.781
Cerebrospinal Fluid Parameters, Median (IQR)				
Protein (mg/L)	805.50 (510.25, 1383.00)	187.50 (141.00, 269.00)	< 0.001^d^; Z=-13.736	< 0.05
Glucose (mmol/L)	2.64 (1.85, 3.25)	3.60 (3.28, 4.27)	< 0.001^d^; Z=-11.058	< 0.05
Chloride (mmol/L)	117.00 (114.00, 120.00)	120.95 (117.00, 124.00)	< 0.001^d^; Z=-5.865	< 0.05
White Blood Cell (×10⁶/L)	0.08 (0.02, 0.47)	0.00 (0.00, 0.02)	< 0.001^d^; Z=-11.513	< 0.05
Segmented Neutrophil (×10⁶/L)	15.15 (2.25, 64.75)	0.00 (0.00, 2.00)	< 0.001^d^; Z=-11.733	< 0.05
Monocyte (×10⁶/L)	26.50 (10.25, 59.75)	4.00 (3.00, 15.00)	< 0.001^d^; Z=-8.588	< 0.05

*IQR* Interquartile range, *CNS* Central nervous system, *PICU* Pediatric intensive care unit

Categorical variables are presented as *n* (%). Continuous variables not following a normal distribution are presented as median (interquartile range)

*P* values are for comparisons between the Bacterial and nonbacterial CNS infection groups

Values < 0.05 were considered statistically significant after correction

Statistical Methods: ᵃChi-square test; ᵇFisher’s exact test; ^d^Mann-Whitney U test

*Adjusted *P* values were calculated using the Benjamini-Hochberg FDR method

Among all 422 cases, fever was the predominant presenting symptom (90.8%), followed by seizure (39.6%), rash (13.7%), and headache (12.3%). Clinical presentations differed notably between groups. Although fever was highly prevalent in both groups, nonbacterial infections were associated with a higher frequency of neurological symptoms, including headache, seizure, and impaired consciousness (all *P* < 0.001). No significant differences were found for other symptoms, such as vomiting, rash, and cough. The associated clinical profiles were also distinct. Clinical bacterial infections showed significantly higher rates of sepsis, pneumonia, anemia, and prematurity (all *P* < 0.001), as well as exclusive occurrences of hydrocephalus and encephalomalacia (*P* < 0.001). In contrast, hand, foot and mouth disease and herpangina were observed only in the clinical nonbacterial group (*P* < 0.001). Rates of respiratory failure did not differ significantly.

Laboratory findings in blood and CSF provided effective discriminators between the two groups. Hematologically, clinical bacterial infections were characterized by elevated inflammatory markers (WBC, neutrophils, lymphocytes, platelets, CRP, PCT) but lower total protein and albumin (all *P* < 0.001 except neutrophils, *P* = 0.019). CSF analysis revealed a distinct profile in the bacterial group: higher protein, lower glucose and chloride, and significantly elevated cell counts (WBC, segmented neutrophils, monocytes; all *P* < 0.001). There was no statistically significant difference in the distribution of clinical outcomes between the two groups (all *P* > 0.05), with comparable cured and improved rates of 95.5% (126/132) in the clinical bacterial group and 93.1% (270/290) in the nonbacterial group. A sensitivity analysis excluding 13 preterm neonates yielded consistent findings. All key differences between bacterial and nonbacterial groups remained statistically significant, with no change in significance for any variable (Supplementary Table S1).

### Diagnostic performance of biomarkers

ROC analysis was performed on biomarkers that showed significant differences between bacterial and nonbacterial groups in univariate analysis. The results are summarized in Table 3.

**Table 3:** Receiver operating characteristic analysis of biomarkers for distinguishing bacterial from nonbacterial CNS infections

Biomarker	AUC	95% CI	*P* value	Cut-off	Sensitivity (%)	Specificity (%)
Blood Parameters						
Albumin (g/L)	0.869	0.835–0.903	< 0.001	≤ 34.6	50.8	93.8
Total protein (g/L)	0.817	0.769–0.865	< 0.001	≤ 54.9	50.0	94.1
CRP (mg/L)	0.801	0.752–0.849	< 0.001	5.96	72.7	67.9
WBC (×10⁹/L)	0.688	0.632–0.744	< 0.001	8.98	75.8	54.1
PCT (ng/mL)	0.682	0.625–0.738	< 0.001	0.285	55.3	72.4
Lymphocyte (×10⁹/L)	0.681	0.625–0.736	< 0.001	2.28	75.0	51.7
Platelet (×10⁹/L)	0.653	0.587–0.719	< 0.001	250.5	72.7	36.6
Neutrophil (×10⁹/L)	0.571	0.509–0.633	0.019	5.96	50.0	62.1
CSF Parameters						
Protein (mg/L)	0.917	0.887–0.947	< 0.001	327	87.1	84.5
WBC (×10⁶/L)	0.837	0.795–0.879	< 0.001	0.015	76.5	74.8
Glucose (mmol/L)	0.836	0.787–0.884	< 0.001	≤ 2.8	62.1	95.5
Segmented neutrophil (×10⁶/L)	0.833	0.789–0.878	< 0.001	3.5	72.7	80.7
Monocyte (×10⁶/L)	0.759	0.712–0.807	< 0.001	9.5	80.3	69.7
Chloride (mmol/L)	0.678	0.622–0.733	< 0.001	≤ 117	41.7	80.3

*AUC* area under the curve, *CI* confidence interval, *CRP* C-reactive protein, *PCT* procalcitonin, *CSF* cerebrospinal fluid, *WBC* white blood cell

Cut-off values were determined using the Youden index

Among blood parameters, albumin (AUC = 0.869) and total protein (AUC = 0.817) demonstrated good discriminative ability with high specificity (> 93%) but relatively low sensitivity (~ 50%). CRP showed good performance (AUC = 0.801), with an optimal cut-off of 5.96 mg/L (sensitivity 72.7%, specificity 67.9%). PCT showed modest performance (AUC = 0.682), with an optimal cut-off of 0.285 ng/mL (sensitivity 55.3%, specificity 72.4%). Peripheral WBC (AUC = 0.688), lymphocyte (AUC = 0.681), and platelet (AUC = 0.653) showed limited diagnostic value, while neutrophil (AUC = 0.571) had poor discriminative ability.

Among CSF parameters, CSF protein demonstrated excellent discriminative ability (AUC = 0.917), followed by CSF WBC (AUC = 0.837) and CSF glucose (AUC = 0.836). Notably, CSF glucose showed exceptionally high specificity (95.5%) at a cut-off of ≤ 2.8 mmol/L. CSF segmented neutrophil (AUC = 0.833) and CSF monocyte (AUC = 0.759) also showed good to moderate performance, while CSF chloride (AUC = 0.678) showed modest discriminative ability with high specificity (80.3%).

### Etiological distribution

**Fig. 2:**
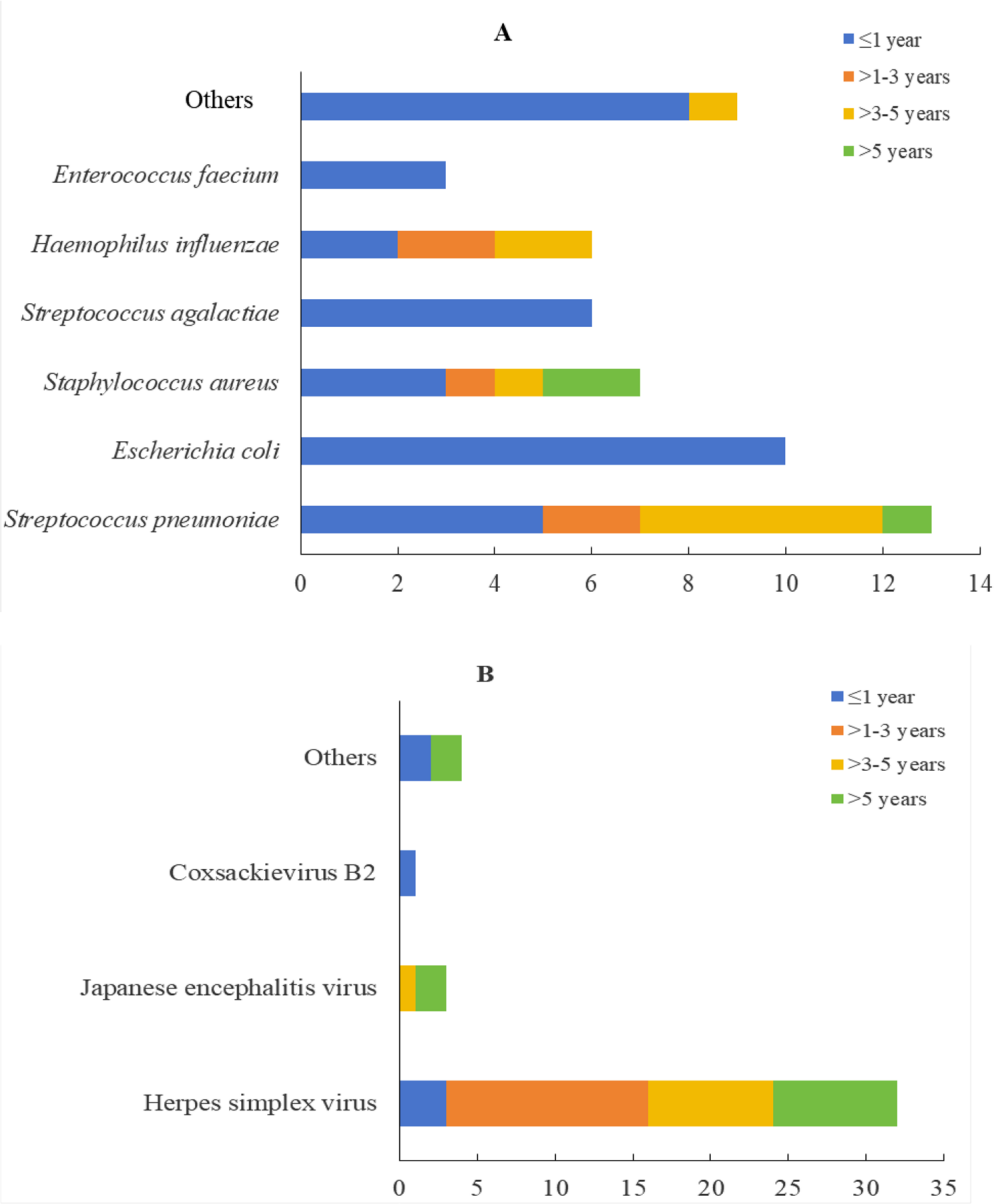
**A–B** Composition of identified pathogens in children with central nervous system infections in Shandong Province, 2020–2024. **A** 54 bacterial cases, **B** 40 viral cases

Pathogens of the 94 CNS infection cases (54 bacterial and 40 viral) were identified through a combination of conventional culture, mNGS, and serological testing. The etiological spectrum is detailed in Table 4, and the age distribution of these cases is shown in Fig. 2. The predominant pathogen was *S. pneumoniae* (13 cases), followed by *Escherichia coli* (*E. coli*; 10 cases), *Staphylococcus aureus* (*S. aureus*; 7 cases), *Streptococcus agalactiae* (*S. agalactiae*; 6 cases), and *H. influenzae* (6 cases). Notable antimicrobial-resistant organisms identified included 4 cases of methicillin-resistant *S. aureus* (MRSA), 3 cases of extended-spectrum beta-lactamase (ESBL)-producing *E. coli*, 1 case of ESBL-producing *Klebsiella pneumoniae*, and one case of carbapenem-resistant *Acinetobacter baumannii* complex (CRAB). Confirmed bacterial CNS infections demonstrated distinct epidemiological features, with an overwhelming predominance in infants ≤ 1 year (37/54, 68.5%). All *S. agalactiae* (6/6) and *E. coli* (10/10) cases occurred exclusively in this age group. Clinical severity was substantial, with 30/54 patients (55.6%) requiring PICU admission, particularly notable in *S. pneumoniae* (10/13).

**Table 4:** Etiological spectrum and clinical severity in children with central nervous system infections

Etiology	All (*n* = 94)	Detection method	Co-detection by mNGS*	PICU admission, *n* (%)	Ward Management, *n* (%)
Bacterial CNS Infections					
* Streptococcus pneumoniae*	13	CSF culture: 6, Blood culture: 1, CSF mNGS: 5, CSF culture and mNGS: 1	—	10 (76.9)	3 (23.1)
* Escherichia coli*	10	CSF culture: 4, Blood culture: 4, CSF mNGS: 2	*Enterococcus faecium*: 1	4 (40.0)	6 (60.0)
* Staphylococcus aureus*	7	CSF culture: 2, Blood culture: 5	—	3 (42.9)	4 (57.1)
* Streptococcus agalactiae*	6	CSF culture: 3, CSF mNGS: 2, CSF culture and mNGS: 1	*Schizophyllum commune*: 1	2 (33.3)	4 (66.7)
* Haemophilus influenzae*	6	CSF culture: 2, CSF mNGS: 3, CSF culture and mNGS: 1	*Pseudomonas* spp.: 1; Middelburg virus I: 1; Torque teno virus and Coxsackievirus A4: 1	4 (66.7)	2 (33.3)
* Enterococcus faecium*	3	CSF culture: 2, CSF mNGS: 1	Torque teno virus and Human herpesvirus 4: 1	2 (66.7)	1 (33.3)
* Listeria monocytogenes*	1	CSF mNGS: 1	—	0 (0.0)	1 (100.0)
* Klebsiella pneumoniae*	2	CSF culture: 1, Blood culture: 1	—	2 (100.0)	0 (0.0)
* Pseudomonas aeruginosa*	2	CSF mNGS: 2	*Stenotrophomonas maltophilia*: 1; *Stenotrophomonas maltophilia* and *Pseudomonas stutzeri*: 1	1 (50.0)	1 (50.0)
* Streptococcus* spp.	2	CSF mNGS: 2	*Staphylococcus* spp. and *Mycoplasma* spp.: 1	1 (50.0)	1 (50.0)
* Acinetobacter baumannii complex*	1	CSF culture: 1	*Stenotrophomonas maltophilia* (culture): 1	1 (100.0)	0 (0.0)
* Streptococcus anginosus*	1	CSF mNGS: 1	*Prevotella bivia*, *Prevotella disiens*, *Peptostreptococcus anaerobius*, *Bacteroides thetaiotaomicron*, and *Bilophila wadsworthia*: 1	0 (0.0)	1 (100.0)
Viral CNS Infections					
Herpes simplex virus	32	CSF mNGS: 6, Serology: 26	—	12 (37.5)	20 (62.5)
Japanese encephalitis virus	3	CSF mNGS: 1, Serology: 2	—	3 (100.0)	0 (0.0)
Coxsackievirus B2	1	CSF mNGS: 1	—	0 (0.0)	1 (100.0)
Human herpesvirus 3	1	CSF mNGS: 1	human herpesvirus 4: 1	1 (100.0)	0 (0.0)
Human herpesvirus 7	1	CSF mNGS: 1	—	1 (100.0)	0 (0.0)
Parechovirus^†^	1	CSF mNGS: 1	—	0 (0.0)	1 (100.0)
Cytomegalovirus	1	CSF mNGS: 1	—	0 (0.0)	1 (100.0)

*CNS* Central nervous system, *PICU* Pediatric intensive care unit, *mNGS* metagenomic next-generation sequencing, *CSF* cerebrospinal fluid

*Co-detections were identified by mNGS unless otherwise indicated. “(culture)” denotes detection by CSF culture

^†^Parechovirus was identified by mNGS at an external hospital prior to admission

Herpes simplex virus (HSV) was the most frequently detected pathogen, identified in 32 cases. HSV was associated with encephalitis across a wide age range. JEV was detected in three children older than 3 years (1 by mNGS, 2 by serology), all of whom presented with severe encephalitis requiring PICU admission. The overall PICU admission rate for this viral cohort was 42.5% (17/40), underscoring the substantial disease burden.

Among the 54 confirmed bacterial cases, 21 (38.9%) were detected by CSF culture alone, 11 (20.4%) by blood culture alone, 19 (35.2%) by CSF mNGS alone, and 3 (5.6%) by both CSF culture and mNGS. For viral infections, 12 cases (30.0%) were detected by CSF mNGS alone, 28 (70.0%) by serology alone. Co-detections were identified in 12 of the 94 cases, of which 11 (91.7%) were detected by mNGS and 1 by CSF culture. The co-detections included 6 bacterial-bacterial co-detections, 4 bacterial-viral co-detections, 1 bacterial-fungal co-detection and 1 viral-viral co-detection. One case of mixed anaerobic infection identified by mNGS has been reported separately [13].

### Characteristics of patients in the PICU

Among 422 patients, 175 (41.5%) required PICU admission, while 247 (58.5%) were managed in general wards (Table 5). No significant differences existed in sex distribution, age, or age distribution between the two groups. However, the median length of hospital stay was significantly longer in PICU patients (16.00 days) than in ward patients (14.00 days; *P* = 0.025). Children admitted to the PICU had significantly worse clinical outcomes compared with those managed in the general ward (*P* < 0.001). The overall cure and improvement rate was substantially lower in the PICU group (86.85%) than in the ward group (98.78%, *P* < 0.001), while the rate of not healed outcomes was significantly higher in the PICU group (13.14%) than in the ward group (1.21%, *P* < 0.001). However, fever, rash, and length of hospital stay were not significantly associated after FDR correction (Table 5).

**Table 5:** Comparison of clinical profiles in PICU-admitted and ward-managed children with central nervous system infections

Characteristics	PICU Admission (*n* = 175)	Ward Management (*n* = 247)	*P* Value	Adjusted *P* Value*
Male Sex, *n* (%)	104 (59.4)	141 (57.1)	0.631ᵃ	0.871
Length of Hospital Stay, days, Median (IQR)	16.00 (11.00, 24.00)	14.00 (10.00, 21.00)	0.025^d^; Z=-2.237	0.066
Age in years, Median (IQR)	2.46 (0.90, 5.30)	2.28 (0.24, 5.54)	0.191^d^; Z=-1.309	0.326
Age Group, *n* (%)			0.634ᵃ	0.836
≤ 1 year	46 (26.3)	78 (31.6)		
> 1–3 years	48 (27.4)	64 (25.9)		
> 3–5 years	32 (18.3)	34 (13.8)		
> 5–7 years	21 (12.0)	33 (13.4)		
> 7–10 years	13 (7.4)	22 (8.9)		
> 10 years	15 (8.6)	16 (6.5)		
Clinical characteristics, *n* (%)				
Fever	153 (87.4)	230 (93.1)	0.047ᵃ	0.105
Headache	12 (6.9)	40 (16.2)	0.004ᵃ	0.015
Seizure	88 (50.3)	79 (32.0)	< 0.001ᵃ	< 0.05
Vomiting	26 (14.9)	17 (6.9)	0.008ᵃ	0.023
Rash	17 (9.7)	41 (16.6)	0.043ᵃ	0.104
Cough	21 (12.0)	24 (9.7)	0.454ᵃ	0.693
Somnolence	15 (8.6)	3 (1.2)	< 0.001ᵇ	< 0.05
Impaired Consciousness	39 (22.3)	0 (0.0)	< 0.001ᵇ	< 0.05
Neck Stiffness	2 (1.1)	4 (1.6)	1.000ᵇ	1.000
Jaundice	2 (1.1)	10 (4.0)	0.134ᵇ	0.259
Gait Disturbance	3 (1.7)	4 (1.6)	1.000ᵇ	1.000
Sepsis	44 (25.1)	64 (25.9)	0.859ᵃ	1.000
Pneumonia	94 (53.7)	67 (27.1)	< 0.001ᵃ	< 0.05
Anemia	19 (10.9)	28 (11.3)	0.878ᵃ	1.000
Hand, Foot and Mouth Disease	8 (4.6)	22 (8.9)	0.088ᵃ	0.182
Herpangina	13 (7.4)	29 (11.7)	0.145ᵃ	0.263
Prematurity	4 (2.3)	9 (3.6)	0.571ᵇ	0.828
Respiratory Failure	34 (19.4)	1 (0.4)	< 0.001ᵇ	< 0.05
Hydrocephalus	9 (5.1)	7 (2.8)	0.301ᵇ	0.485
Encephalomalacia	3 (1.7)	4 (1.6)	1.000ᵇ	1.000
Subdural Effusion	1 (0.6)	1 (0.4)	1.000ᵇ	1.000
Non-traumatic Intracranial Hemorrhage	1 (0.6)	1 (0.4)	1.000ᵇ	1.000
Prognosis *n* (%)				
Cured	101 (57.7)	200 (81.0)	< 0.001ᵃ	< 0.05
Improved	51 (29.1)	44 (17.8)	0.006ᵃ	0.019
Not healed	23 (13.1)	3 (1.2)	< 0.001ᵇ	< 0.05

*IQR* Interquartile range, *PICU* Pediatric intensive care unit

Categorical variables are presented as *n* (%). Continuous variables not following a normal distribution are presented as median (interquartile range)

*P* values are for comparisons between the Severe and Non-Severe groups

Values < 0.05 were considered statistically significant after correction

Statistical Methods: ᵃChi-square test; ᵇFisher’s exact test; ^d^Mann-Whitney U test

*Adjusted *P* values were calculated using the Benjamini-Hochberg FDR method

### Multivariate analysis of PICU admission predictors

To identify independent predictors of PICU admission, binary logistic regression was performed with PICU admission as the dependent variable. The model included age, length of hospital stay, and clinical features (fever, headache, seizure, vomiting, rash, somnolence, pneumonia, respiratory failure) as covariates. Impaired consciousness was excluded from the model because it perfectly predicted PICU admission (all 39 patients with impaired consciousness were admitted to the PICU). Collinearity diagnostics showed no evidence of significant multicollinearity, with all VIF values below 2.0 (Table 6).

**Table 6:** Multivariate analysis of factors associated with PICU admission

Variable	Adjusted OR	95% CI	*P* value	VIF
Respiratory failure	39.76	5.16–306.26	< 0.001	1.313
Somnolence	15.03	3.49–64.64	< 0.001	1.038
Vomiting	3.93	1.66–9.30	0.002	1.109
Seizure	2.53	1.54–4.15	< 0.001	1.208
Pneumonia	2.51	1.54–4.08	< 0.001	1.174
Age (years)	1.09	1.01–1.18	0.023	1.488
Length of Hospital Stay (days)	1.02	1.00–1.05	0.032	1.139
Rash	0.70	0.36–1.39	0.307	1.038
Fever	0.52	0.24–1.12	0.096	1.020
Headache	0.19	0.07–0.53	0.001	1.578

*OR* odds ratio, *CI* confidence interval, *VIF* variance inflation factor, *PICU* pediatric intensive care unit

Binary logistic regression was performed with PICU admission as the dependent variable. Impaired consciousness was excluded from the multivariate model because it perfectly predicted PICU admission (all 39 patients with impaired consciousness were admitted to the PICU); with a VIF of 1.387. The Hosmer-Lemeshow test indicated good model fit (χ² = 2.36, df = 8, *P* = 0.968)

After adjustment, respiratory failure (aOR = 39.76, 95% CI: 5.16–306.26, *P* < 0.001), somnolence (aOR = 15.03, 95% CI: 3.49–64.64, *P* < 0.001), vomiting (aOR = 3.93, 95% CI: 1.66–9.30, *P* = 0.002), seizure (aOR = 2.53, 95% CI: 1.54–4.15, *P* < 0.001), pneumonia (aOR = 2.51, 95% CI: 1.54–4.08, *P* < 0.001), age (aOR = 1.09, 95% CI: 1.01–1.18, *P* = 0.023), and length of hospital stay (aOR = 1.02, 95% CI: 1.00–1.05, *P* = 0.032) were identified as independent predictors of PICU admission.

In contrast, headache was associated with lower odds of PICU admission (aOR = 0.19, 95% CI: 0.07–0.53, *P* = 0.001), while fever and rash were not independently associated after adjustment (both *P* > 0.05). The Hosmer-Lemeshow test indicated good model fit (χ² = 2.36, df = 8, *P* = 0.968) (Table 6).

### Sensitivity analysis of the impact of the COVID-19 pandemic

To assess potential temporal confounding by the COVID-19 pandemic, we analyzed the annual distribution of CNS infections (Supplementary Fig. S1). Total cases decreased in 2022 (*n* = 69) compared with 2021 (*n* = 131), then rose to 89 in 2023. The proportion of bacterial infections (29.0%–36.6%) and PICU admission rates (33.7%–46.3%) remained stable across the study period.

## Discussion

This retrospective study of 422 pediatric patients provides a comprehensive analysis of CNS infections over a five-year period at Shandong Regional Children’s Medical Center. By integrating detailed demographic, clinical and laboratory findings, our results offer a practical clinical guide to accelerate diagnosis, direct targeted treatment, and identify high-risk children for early intervention.

The substantially prolonged median hospital stay of 15 days, more than double the hospital’s average of approximately 7 days, highlights the significant healthcare burden and complexity associated with these infections. This extended duration, particularly in meningitis and meningoencephalitis (both 23 days), reflects the need for protracted treatment, monitoring for complications, and recovery time [14]. The median age of 2.38 years underscores the heightened vulnerability of toddlers and preschoolers to severe CNS infections. Notably, the distribution varied markedly by diagnosis: meningitis and meningoencephalitis overwhelmingly affected infants (median ages 0.23 and 0.18 years, respectively), while encephalitis patients were significantly older (median 3.89 years). This pattern aligns with the fact that children under 5 years of age are at the highest risk due to immature immune systems and compromised blood-brain barriers, which increase their susceptibility to severe disease.

This study reveals that fever (90.8%) and seizure (39.6%) are the most common presenting symptoms in pediatric CNS infections. In contrast, classic neurological signs such as headache and neck stiffness, which are frequently observed in adults with CNS infections, were uncommon in this pediatric population [3, 15]. These findings highlight the limited diagnostic reliability of adult-derived traditional signs in children and underscore the need for a high index of clinical suspicion to ensure timely diagnosis in this population.

The rapid differentiation between clinical bacterial and nonbacterial pediatric CNS infections is critical for timely antimicrobial therapy. Notably, our study revealed distinct and clinically useful characteristics between clinical bacterial and nonbacterial pediatric CNS infection groups. Unlike previous studies [16], we observed a higher incidence of headache, impaired consciousness, and seizure in the nonbacterial CNS infection group. This discrepancy may be attributed to the younger age of the clinical bacterial group (median 0.22 years), where infants are unable to verbalize symptoms such as headache. Regarding complications, the clinical bacterial group demonstrated a significantly higher incidence of pneumonia and sepsis (*P* < 0.001), probably because pathogens such as *S. pneumoniae*, *E. coli* and *S. aureus* are capable of causing systemic and pulmonary infections, leading to concurrent sepsis and pneumonia [17–19]. Hydrocephalus and encephalomalacia were more prevalent in the clinical bacterial group (both *P* < 0.001). Hydrocephalus, a severe complication of neonatal bacterial meningitis (9%–35%), arises from inflammatory obstruction of CSF pathways and carries the risk of irreversible brain injury without prompt intervention [20], while encephalomalacia often results from septic infarction or severe inflammation, and is a strong predictor of permanent neurological sequelae [14]. Anemia and prematurity were significantly more common in the clinical bacterial infection group (both *P* < 0.001). These conditions likely increase susceptibility to bacterial pathogens and indicate a vulnerable host state. In contrast, the lack of significant difference in the rate of respiratory failure between groups suggests that they may be more influenced by individual patient factors than by the specific type of infecting pathogen. Additionally, hand, foot and mouth disease and herpangina occurred exclusively in the nonbacterial group. These conditions are typically caused by enteroviruses, which are common pathogens known to cause both these specific syndromes and a significant proportion of viral CNS infections [21]. Thus, systematic evaluation of symptoms, unique clinical manifestations, and complications facilitates reliable differentiation between clinical bacterial and nonbacterial CNS infections.

In this study, CSF analysis revealed a characteristic profile for clinical bacterial CNS infections. The clinical bacterial group exhibited markedly elevated protein (805.50 versus 187.50 mg/L; *P* < 0.001), significantly lower glucose (2.64 versus 3.60 mmol/L; *P* < 0.001), and a profound neutrophilic pleocytosis with higher segmented neutrophil (15.15 versus 0.00 × 10⁶/L; *P* < 0.001) and monocyte counts (26.50 versus 4.00 × 10⁶/L; *P* < 0.001). Additionally, CSF chloride levels were significantly lower (117.00 versus 120.95 mmol/L; *P* < 0.001). Collectively, these findings corroborate the classic biochemical profile of bacterial CNS infection supported by the literature [8]. However, in our cohort, 22.7% (30/132) of confirmed bacterial CNS infections presented with normal CSF protein, underscoring that conventional CSF tests alone are therefore insufficient for clinical decision-making. ROC analysis further confirmed the diagnostic utility of these CSF parameters. CSF protein demonstrated excellent discriminative ability (AUC = 0.917), with an optimal cut-off of 327 mg/L (sensitivity 87.1%, specificity 84.5%). CSF WBC (AUC = 0.837) and CSF glucose (AUC = 0.836) also showed good performance, with CSF glucose achieving exceptionally high specificity (95.5%) at a cut-off of ≤ 2.8 mmol/L. CSF segmented neutrophil (AUC = 0.833) and CSF monocyte (AUC = 0.759) showed good to moderate performance, while CSF chloride (AUC = 0.678) showed modest discriminative ability with high specificity (80.3%). These findings support the continued use of CSF parameters as reliable diagnostic markers for bacterial CNS infections.

Biologically, CRP acts as a sensitive acute-phase marker of systemic inflammation for bacterial infection evaluation [22], while PCT, as a calcitonin precursor, rises within hours of bacterial infection and is relatively unaffected by antibiotics, underscoring its clinical utility for differential diagnosis and therapeutic monitoring [23]. In our cohort, clinical bacterial infections were characterized by marked elevations in serum CRP and PCT. The median levels were substantially higher in bacterial cases compared with nonbacterial infections (CRP: 36.70 vs. 3.20 mg/L; PCT: 0.34 vs. 0.13 ng/mL; both *P* < 0.001), consistent with prior studies [8, 24]. ROC analysis confirmed that CRP had good diagnostic performance (AUC = 0.801), with an optimal cut-off of 5.96 mg/L (sensitivity 72.7%, specificity 67.9%). In contrast, PCT showed only modest performance in our cohort (AUC = 0.682). Previous studies have reported higher diagnostic accuracy for PCT in bacterial CNS infections, with sensitivities of 89–95% and specificities of 84–100% at cut-offs of 0.28–0.5 ng/mL in children [8, 24]. However, a meta-analysis of community-acquired pneumonia reported an overall sensitivity of 55% and specificity of 76% [25], suggesting that PCT performance varies across populations and clinical settings. Given its modest overall performance in our cohort, PCT should not be used alone, and its interpretation must be combined with clinical and CSF findings.

Additionally, serum albumin and total protein, which were significantly lower in the bacterial group, showed good discriminative ability (AUC = 0.869 and 0.817, respectively), with high specificity (> 93%) but relatively low sensitivity (~ 50%). This finding suggests that hypoalbuminemia and hypoproteinemia are relatively common in children with bacterial CNS infections, potentially reflecting systemic metabolic derangement associated with severe infection. Furthermore, we found that the significantly higher peripheral WBC, neutrophil, lymphocyte, and platelet counts in the clinical bacterial group (all *P* < 0.05) further support that bacterial infections trigger a more pronounced systemic inflammatory response. However, ROC analysis showed that these peripheral markers had limited diagnostic value, with AUC values ranging from 0.571 to 0.688. Notably, peripheral WBC (AUC = 0.688) and lymphocyte (AUC = 0.681) showed modest performance, while neutrophil (AUC = 0.571) had poor discriminative ability.

Therefore, we strongly propose that CSF profiles be interpreted in combination with blood biomarkers (CRP, albumin, and total protein). This combined diagnostic approach provides a practical strategy to guide empirical antimicrobial therapy while awaiting definitive microbiological results.

Recognizing specific pathogens is critical, as it directly determines appropriate therapeutic interventions. In our cohort, the predominant bacterial agents identified were *S. pneumoniae* (*n* = 13) and *E. coli* (*n* = 10), which exhibited a clear age-dependent distribution. This pattern aligns with global epidemiological data, confirming *S. pneumoniae* as a leading cause of bacterial CNS infections [15, 26]. Within our cohort, *S. pneumoniae* cases were predominantly observed in children over 1 year of age (8/13 cases). A critical revelation was that infants aged ≤ 1 year bore an overwhelming burden, accounting for 68.5% (37/54) of confirmed bacterial CNS infections. Within this vulnerable population, *E. coli* (*n* = 10) and *S. agalactiae* (*n* = 6) were the leading causes, highlighting an age restriction consistent with reports from other regions in China [27, 28]. The detection of antimicrobial-resistant organisms (notably MRSA, ESBL-producing Enterobacteriaceae, and CRAB) within the confirmed bacterial cases highlights a significant and growing challenge for empirical antimicrobial therapy in pediatric CNS infections. This finding necessitates heightened vigilance and may prompt consideration of broader-spectrum or pathogen-directed initial regimens in high-risk settings, especially when local epidemiology or clinical severity suggests such a threat. Disease severity was substantial, with 55.6% (30/54) of confirmed bacterial cases requiring PICU admission. *S. pneumoniae* was most strongly associated with critical illness, with 76.9% (10/13) of its cases requiring intensive care, a finding consistent with its established high virulence [29, 30]. Literature has shown that *L. monocytogenes* can cause CNS infections in neonates, adults, and immunocompetent patients [5]. We detected *L. monocytogenes* in the CSF of a previously healthy 4-year-old boy using mNGS, which suggests that clinicians should maintain suspicion for such infections even in previously healthy children. Similarly, Deniz et al. reported the detection of *L. monocytogenes* in CSF cultures from a previously healthy 16-year-old patient [16].

For viral etiologies, HSV was the most common viral pathogen in our cohort, detected by mNGS and serological testing as clinically indicated. Consistent with previous studies, HSV remains a leading global cause of pediatric encephalitis, known for its severe, life-threatening potential in childhood and the challenge of lifelong neuronal latency, which demands optimized management strategies [31, 32]. In contrast, JEV presented with a distinct and severe clinical phenotype, occurring only in children older than 3 years. All three cases required PICU admission, highlighting the significant regional disease burden of this neurotropic arbovirus [33].

The application of mNGS was highly valuable in our cohort, particularly for pathogen detection in culture-negative cases and co-infections. Among 54 confirmed bacterial cases, mNGS alone detected 19 (35.2%). Furthermore, 11 of 12 co-detections (91.7%) were identified by mNGS, underscoring its superiority in detecting mixed infections. Notably, *Candida glabrata* alone was detected by mNGS with low reads in one patient who presented with typical bacterial meningitis and improved with antibiotics alone, highlighting that mNGS results should be interpreted in conjunction with clinical presentation and treatment response. As this was a retrospective study, mNGS was not routinely performed in all patients but was used selectively based on clinical necessity. Given the high proportion of critically ill children requiring a definitive diagnosis, mNGS effectively complemented conventional culture methods and significantly improved diagnostic yield for pediatric CNS infections [34].

Early identification of children at risk of clinical deterioration is essential for improving outcomes. The 41.5% PICU admission rate in our cohort underscores the substantial burden of severe CNS infections. Our multivariate analysis identified respiratory failure (aOR = 39.76), somnolence (aOR = 15.03), vomiting (aOR = 3.93), seizure (aOR = 2.53), pneumonia (aOR = 2.51), age (aOR = 1.09), and length of hospital stay (aOR = 1.02) as independent predictors of PICU admission. Notably, respiratory failure exhibited an extremely wide 95% confidence interval (5.16–306.26), likely resulting from the limited sample size and low number of outcome events. Accordingly, this estimate should be interpreted with considerable caution in clinical practice. Although statistically significant, the clinical significance of this small effect size for age is limited. This finding likely reflects that older children may present with more recognizable neurological symptoms that prompt earlier PICU referral, rather than a true age-related increase in disease severity. Impaired consciousness was also strongly associated with PICU admission but was excluded from the multivariate model due to complete separation (all 39 patients with impaired consciousness were admitted to the PICU). Specifically, impaired consciousness and seizure emerge as critical clinical predictors for PICU need, aligning with prior reports [16, 35, 36]. Furthermore, somnolence may serve as an early, subtler indicator of emerging neurological compromise, preceding more overt impaired consciousness.

We also demonstrate that pneumonia and respiratory failure are highly prevalent systemic complications in PICU cases, indicating that multi-organ dysfunction is a key driver of severity. Interestingly, headache was associated with lower odds of PICU admission (aOR = 0.19), which may reflect that children with headache alone often present with milder disease. Fever and rash were not independently associated after adjustment, suggesting that these symptoms are less specific for severe disease. Notably, differences in the prevalence of symptoms like vomiting, compared with other studies [16], suggest that clinical presentations may vary based on etiology and population characteristics.

Consistent with the finding that a 94.4% overall cure and improvement rate was reported for children with community-acquired bacterial meningitis (CABM) in Zhejiang Province between 2019 and 2020 [28], our study similarly demonstrated favorable overall clinical outcomes in pediatric CNS infections, with a combined cure and improvement rate of 93.9%. Notably, no significant prognostic differences were observed between clinical bacterial and nonbacterial subgroups (95.45% versus 93.10%, *P* > 0.05), and our data further analyzed the cure and improvement rates in children admitted to the PICU versus those managed in the general ward, which were 86.85% and 98.78%, respectively. This study offers valuable clinical insights for physicians: age-specific pathogens (*E. coli*, *S. agalactiae*) should guide antibiotic selection in infants. CSF protein, CSF WBC, and CSF glucose provide reliable diagnostic performance. Serum albumin (cut-off ≤ 34.6 g/L, specificity 93.8%), total protein (cut-off ≤ 54.9 g/L, specificity 94.1%), and CRP (cut-off 5.96 mg/L, AUC = 0.801) demonstrate good discriminative ability, while peripheral WBC shows moderate performance (AUC = 0.688). In contrast, PCT shows modest performance (AUC = 0.682) and should be interpreted with caution. Impaired consciousness, respiratory failure, somnolence, vomiting, seizure, and pneumonia were independent predictors of PICU admission and should all be considered in clinical risk assessment. Integrating these clinical, laboratory, and pathogen findings is essential for accurate diagnosis and improved outcomes.

This study has several limitations. First, the nonbacterial group combined viral infections with cases of unknown etiology. Cases of unknown etiology could include undetected infections (particularly in the context of prior antibiotic use) or other non-infectious conditions. While this approach reflects clinical practice where definitive pathogens are not always identified, it may introduce heterogeneity. Second, HSV diagnoses in some cases relied on serological testing, which has limited sensitivity and specificity. Given that mNGS and serological testing were selectively used as clinically indicated, while PCR or multiplex PCR panels were not routinely performed, our data may be subject to selection bias toward more severe disease and may not represent the full spectrum of CNS infections. Third, the impact of antibiotic pretreatment on CSF culture negativity was not assessed, and long-term neurodevelopmental follow-up was not performed, limiting our ability to assess both diagnostic yield and permanent neurological sequelae. Fourth, the use of mNGS was targeted, most likely applied to more severe or diagnostically complex cases, which could introduce selection bias into the etiological analysis. Additionally, the study period overlapped with the COVID-19 pandemic, which may have introduced temporal confounding. Fifth, as a single-center study conducted at a tertiary referral hospital, our findings may be subject to tertiary-referral bias and may not be fully generalizable to primary or secondary care settings.

## Conclusion

This five-year cohort study provides critical insights for the early identification of clinical bacterial CNS infections and children at high risk of PICU admission. Our study demonstrated that normal CSF examination results and negative CSF culture do not exclude bacterial CNS infections, and the integration of clinical, etiological, and laboratory features for comprehensive assessment is therefore essential for accurate diagnosis. CSF parameters demonstrated excellent to good diagnostic performance for distinguishing bacterial from nonbacterial CNS infections. CRP showed good discriminative ability (AUC = 0.801). Serum albumin and total protein (AUC = 0.869 and 0.817, respectively) also performed well, but with sensitivity of only approximately 50%, they should not be used alone for diagnosis to avoid clinical misinterpretation. PCT showed only modest diagnostic performance (AUC = 0.682) in our cohort and should not be relied upon as a standalone test. Impaired consciousness, respiratory failure, somnolence, vomiting, seizure, and pneumonia were independent predictors of PICU admission. Infants ≤ 1 year, constituting a significant proportion of confirmed bacterial CNS infection cases with specific pathogens and atypical manifestations, pose considerable challenges to clinical diagnosis and treatment. mNGS can detect pathogens missed by conventional culture and identify co-infections, serving as a valuable complementary tool in complex or clinically challenging cases. These findings provide a practical framework to guide clinical decision-making and improve outcomes in this vulnerable pediatric population. 

## Supplementary Information

Supplementary Material 1: Table S1 Demographic, clinical and laboratory findings in children clinically diagnosed with bacterial and nonbacterial central nervous system infections after exclusion of (excluding preterm neonates). Fig S1. Annual distribution of central nervous system infection and PICU admission cases, 2020–2024 (Sensitivity analysis). Note: The study period was divided into two phases: the pandemic period (2020–2022) and the post-pandemic period (2023–2024), according to the relaxation of COVID-19 control measures in China.

## Data Availability

The datasets generated and/or analysed during the current study are not publicly available to ensure the privacy of the study participants. They are available from the corresponding author on reasonable request.

## Abbreviations

CNS: Central nervous system
CSF: Cerebrospinal fluid
PICU: Pediatric intensive care unit
CRP: C-reactive protein
PCT: Procalcitonin
WBC: White blood cell
mNGS: Metagenomic next-generation sequencing
*S*. *pneumoniae*: Streptococcus pneumoniae
*E. coli*: *Escherichia coli*
*S. aureus*: *Staphylococcus aureus*
*S. agalactiae*: *Streptococcus agalactiae*
*H. influenzae*: *Haemophilus influenzae*
*E. faecium*: *Enterococcus faecium*
*L. monocytogenes*: *Listeria monocytogenes*
JEV: Japanese encephalitis virus
HSV: Herpes simplex virus
MRSA: Methicillin-resistant *Staphylococcus aureus*
ESBL: Extended-spectrum beta-lactamase
CRAB: Carbapenem-resistant *Acinetobacter baumannii* complex
Benjamini-Hochberg FDR: Benjamini-Hochberg false discovery rate
aOR: Adjusted odds ratios
OR: Odds ratio
CI: Confidence interval
VIF: Variance inflation factor
ROC: Receiver operating characteristic
AUC: Area under the curve
