# Supplementary material for: Clinical and laboratory profiles of acute central nervous system infections in a Chinese pediatric cohort: a five-year retrospective analysis
Source: BMC Pediatr. 2026 Apr 25;26:548. doi: 10.1186/s12887-026-06932-1 (PMC13248359; doi:10.1186/s12887-026-06932-1)
Supplement: Supplementary file 1 — Supplementary Material 1: Table S1 Demographic, clinical and laboratory findings in children clinically diagnosed with bacterial and nonbacterial central nervous system infections after exclusion of (excluding preterm neonates). Fig S1. Annual distribution of central nervous system infection and PICU admission cases, 2020–2024 (Sensitivity analysis). Note: The study period was divided into two phases: the pandemic period (2020–2022) and the post-pandemic period (2023–2024), according to the relaxation of COVID-19 control measures in China. [file 12887_2026_6932_MOESM1_ESM.docx]

**Supplementary Table S1 Demographic, clinical and laboratory findings in children clinically diagnosed with bacterial and nonbacterial central nervous system infections after exclusion of (excluding preterm neonates)**

| **Characteristics** | **Clinical Bacterial CNS Infection (n=120)** | **Clinical Nonbacterial CNS Infection (n=289)** | **P Value** | **Adjusted**  **P Value*** |
| --- | --- | --- | --- | --- |
| Male Sex, n (%) | 65 (54.2) | 173 (59.9) | 0.288ᵃ | 0.370 |
| Length of Hospital Stay, days, Median (IQR) | 23.00 (17.00, 32.00) | 13.00 (9.50, 16.00) | <0.001^d^; Z=-10.598 | <0.05 |
| Age in years, Median (IQR) | 0.23 (0.12, 0.90) | 3.92 (1.91, 6.83) | <0.001^d^; Z=-12.190 | <0.05 |
| **Age Group, n (%)** |  |  | <0.001ᵃ | <0.05 |
| ≤1 year | 91 (75.8) | 20 (6.9) |  |  |
| >1-3 years | 12 (10.0) | 100 (34.6) |  |  |
| >3-5 years | 11 (9.2) | 55 (19.0) |  |  |
| >5-7 years | 2 (1.7) | 52 (18.0) |  |  |
| >7-10 years | 1 (0.8) | 34 (11.8) |  |  |
| >10 years | 3 (2.5) | 28 (9.7) |  |  |
| PICU Admission, n (%) | 49 (40.8) | 122 (42.2) | 0.797ᵃ | 0.854 |
| **Clinical characteristics, n (%)** |  |  |  |  |
| Fever | 112 (93.3) | 261 (90.3) | 0.326ᵃ | 0.408 |
| Headache | 5 (4.2) | 47 (16.3) | 0.001ᵃ | <0.05 |
| Seizure | 23 (19.2) | 142 (49.1) | <0.001ᵃ | <0.05 |
| Vomiting | 11 (9.2) | 32 (11.1) | 0.567ᵃ | 0.638 |
| Rash | 12 (10.0) | 44 (15.2) | 0.162ᵃ | 0.221 |
| Cough | 15 (12.5) | 30 (10.4) | 0.533ᵃ | 0.615 |
| Somnolence | 5 (4.2) | 13 (4.5) | 0.882ᵃ | 0.923 |
| Impaired Consciousness | 2 (1.7) | 37 (12.8) | <0.001ᵇ | <0.05 |
| Neck Stiffness | 4 (3.3) | 2 (0.7) | 0.064ᵇ | 0.103 |
| Jaundice | 3 (2.5) | 7 (2.4) | 1.000ᵇ | 1.000 |
| Gait Disturbance | 0 (0.0) | 7 (2.4) | 0.111ᵇ | 0.156 |
| Sepsis | 85 (70.8) | 16 (5.5) | <0.001ᵃ | <0.05 |
| Pneumonia | 63 (52.5) | 92 (31.8) | <0.001ᵃ | <0.05 |
| Anemia | 28 (23.3) | 11 (3.8) | <0.001ᵃ | <0.05 |
| Hand, Foot and Mouth Disease | 0 (0.0) | 30 (10.4) | <0.001ᵇ | <0.05 |
| Herpangina | 0 (0.0) | 42 (14.5) | <0.001ᵇ | <0.05 |
| Respiratory Failure | 8 (6.7) | 26 (9.0) | 0.437ᵃ | 0.531 |
| Hydrocephalus | 11 (9.2) | 0 (0.0) | <0.001ᵇ | <0.05 |
| Encephalomalacia | 4 (3.3) | 0 (0.0) | 0.007ᵇ | 0.012 |
| Subdural Effusion | 2 (1.7) | 0 (0.0) | 0.086ᵇ | 0.133 |
| Non-traumatic Intracranial Hemorrhage | 2 (1.7) | 0 (0.0) | 0.086ᵇ | 0.129 |
| **Prognosis, n (%)** |  |  |  |  |
| Cured | 80 (66.7) | 209 (72.3) | 0.253^a^ | 0.335 |
| Improved | 34 (28.3) | 60 (20.8) | 0.097^a^ | 0.141 |
| Not healed | 6 (5.0) | 20 (6.9) | 0.469^a^ | 0.555 |
| **Blood Parameters, Median (IQR)** |  |  |  |  |
| White Blood Cell (×10⁹/L) | 12.10 (9.28, 16.81) | 8.69 (6.14, 11.65) | <0.001^d^; Z=-6.440 | <0.05 |
| Neutrophil (×10⁹/L) | 6.66 (3.40, 11.09) | 4.74 (2.71, 7.94) | 0.002^d^; Z=-3.050 | 0.003 |
| Lymphocyte (×10⁹/L) | 3.79 (2.13, 5.71) | 2.21 (1.34, 3.67) | <0.001^d^; Z=-5.539 | <0.05 |
| Platelet (×10⁹/L) | 376.00 (233.50, 514.00) | 275.00 (218.50, 357.50) | <0.001^d^; Z=-4.841 | <0.05 |
| C-reactive protein (mg/L) | 39.00 (3.60, 97.63) | 3.20 (1.40, 8.33) | <0.001^d^; Z=-9.709 | <0.05 |
| Procalcitonin (ng/mL) | 0.49 (0.10, 4.40) | 0.13 (0.06, 0.33) | <0.001^d^; Z=-6.028 | <0.05 |
| Total Protein (g/L) | 55.00 (48.93, 59.68) | 64.00 (59.85, 68.60) | <0.001d; Z=-9.853 | <0.05 |
| Albumin (g/L) | 34.65 (32.00, 37.95) | 41.10 (38.35, 43.10) | <0.001^d^; Z=-11.615 | <0.05 |
| Glucose (mmol/L) | 5.37 (4.80, 6.51) | 5.37 (4.75, 6.32) | 0.713^d^; Z=-0.367 | 0.783 |
| Lactate dehydrogenase (U/L) | 253.00 (204.75, 296.50) | 249.00 (210.00, 307.00) | 0.934^d^; Z=-0.082 | 0.955 |
| **Cerebrospinal Fluid Parameters, Median (IQR)** |  |  |  |  |
| Protein (mg/L) | 720.50 (436.00, 1262.25) | 187.00 (141.00, 266.50) | <0.001^d^; Z=-13.123 | <0.05 |
| Glucose (mmol/L) | 2.67 (2.03, 3.31) | 3.60 (3.29, 4.27) | <0.001^d^; Z=-10.199 | <0.05 |
| Chloride (mmol/L) | 117.00 (114.00, 120.00) | 121.00 (117.00, 124.00) | <0.001^d^; Z=-5.697 | <0.05 |
| White Blood Cell (×10⁶/L) | 0.09 (0.02, 0.62) | 0.00 (0.00, 0.02) | <0.001^d^; Z=-11.183 | <0.05 |
| Segmented Neutrophil (×10⁶/L) | 17.40 (2.00, 69.05) | 0.00 (0.00, 2.00) | <0.001^d^; Z=-11.450 | <0.05 |
| Monocyte (×10⁶/L) | 24.05 (10.00, 56.25) | 4.00 (3.00, 15.00) | <0.001^d^; Z=-7.976 | <0.05 |

Preterm neonates (n=13; 12 in bacterial group, 1 in nonbacterial group) were excluded from this analysis.

*IQR* Interquartile range, *CNS* Central nervous system, *PICU* Pediatric intensive care unit

Statistical Methods: ᵃChi-square test; ᵇFisher's exact test; ^d^Mann-Whitney U test. Categorical variables are presented as n (%). Continuous variables not following a normal distribution are presented as median (interquartile range). P values are for comparisons between the Bacterial and nonbacterial CNS infection groups.

*Adjusted P values were calculated using the Benjamini-Hochberg FDR method. Values <0.05 were considered statistically significant after correction.

**Supplementary Figure S1** Annual distribution of central nervous system infection and PICU admission cases, 2020–2024 (Sensitivity analysis)

Note: The study period was divided into two phases: the pandemic period (2020–2022) and the post-pandemic period (2023–2024), according to the relaxation of COVID-19 control measures in China.
